# Does a code make a difference – assessing the English code of practice on international recruitment

**DOI:** 10.1186/1478-4491-7-33

**Published:** 2009-04-09

**Authors:** James Buchan, Barbara McPake, Kwadwo Mensah, George Rae

**Affiliations:** 1 Queen Margaret University, Edinburgh, UK; 2 Independent Consultant, Yak Aky Management Consultancy Services, Accra, Ghana; 3 Independent Consultant, Nairobi, Kenya

## Abstract

**Background:**

This paper draws from research completed in 2007 to assess the effect of the Department of Health, England, Code of Practice for the international recruitment of health professionals.

The Department of Health in England introduced a Code of Practice for international recruitment for National Health Service employers in 2001. The Code required National Health Service employers not to actively recruit from low-income countries, unless there was government-to-government agreement. The Code was updated in 2004.

**Methods:**

The paper examines trends in inflow of health professionals to the United Kingdom from other countries, using professional registration data and data on applications for work permits. The paper also provides more detailed information from two country case studies in Ghana and Kenya.

**Results:**

Available data show a considerable reduction in inflow of health professionals, from the peak years up to 2002 (for nurses) and 2004 (for doctors). There are multiple causes for this decline, including declining demand in the United Kingdom.

In Ghana and Kenya it was found that active recruitment was perceived to have reduced significantly from the United Kingdom, but it is not clear the extent to which the Code was influential in this, or whether other factors such as a lack of vacancies in the United Kingdom explains it.

**Conclusion:**

Active international recruitment of health professionals was an explicit policy intervention by the Department of Health in England, as one key element in achieving rapid staffing growth, particularly in the period 2000 to 2005, but the level of international recruitment has dropped significantly since early 2006. Regulatory and education changes in the United Kingdom in recent years have also made international entry more difficult.

The potential to assess the effect of the Code in England is constrained by the limitations in available databases. This is a crucial lesson for those considering a global code: without a clear link between explicit objectives of a code, and relevant monitoring capacity, it is not possible to judge the actual impact of a code.

A second message for policy-makers is that attempts to use a single country code in other countries where there are a multiplicity of independent, private-sector health care employers, or where there is a federated political and regulatory structure, will be a much more challenging and complex issue than in England, which has one major public sector health care employer and one national point of entry for regulated health professionals.

Finally, there is a message about the importance of the "visibility" of any recruitment code – for policy-makers, employers and potential recruits. The Department of Health Code has a good level of recognition in the National Health Service, but would benefit from better dissemination in low-income countries, particularly in Africa, together with further consultation on the appropriateness of its provisions in specific countries. To achieve high visibility and recognition of any global code will be a much bigger challenge.

## Background

International recruitment of health professionals has been high on the policy debate agenda in recent years [1] with increasing advocacy for the development of an international code of practice, notably the current draft for a WHO global code. This paper assesses the effect of the first national code, which has been in place in England since 2001 and as such has lessons for current initiatives in other countries and globally. It is based on research commissioned by the Department for International Development (DFID), with support from the Department of Health (DH) in England, to assess the effect of the Department of Health Code of Practice for the international recruitment of health professionals.

As the DH Code was the first country-level code to be developed, there is particular interest in its content and impact. Given current debate about multinational codes, lessons learnt from the impact of the DH Code have policy relevance. This paper reports on an assessment of the effect of the Code in the "destination" country of England and in two "source" countries.

Active international recruitment of health professionals was an explicit policy intervention by the DH in England, as one key element in achieving rapid staffing growth in the National Health Service (NHS), particularly in the period 2000 to 2005 [2]. In the context of this paper, "active" international recruitment occurs when the employer takes the lead to stimulate interest and recruit health staff from another country. With subsequent growth in the numbers of nurses and medical staff beginning to emerge from United Kingdom-based education, and with financial difficulties hitting some NHS trusts in England in 2006, the level of international recruitment has dropped significantly since early 2006. Assessment of the effect of the Code has to take account of these changing labour market circumstances.

The Department of Health in England first attempted to limit the potential negative impact of international recruitment of health professionals in 1999, when it established guidelines that required NHS employers not to target recruitment activities in South Africa and the West Indies [3]. It then introduced a Code of Practice for international recruitment for NHS employers, in 2001 [4].

The Code issued in 2001 required NHS employers not to actively recruit from low-income countries unless there was government-to-government agreement. A full list of these countries was made available to NHS employers in early 2003. The list of countries was developed by the Department of Health in discussion with DFID. In 2007, at the time of the research used in this paper, the list included 154 countries.

Three countries on the list (China, India and the Philippines) had been exempted at the request of their governments, on the basis of bilateral agreements with the United Kingdom government. This exemption meant that active recruitment could take place, as the governments of these countries had endorsed the practice.

The Code was strengthened in 2004 [5] when it was extended to cover recruitment agencies working for NHS employers, temporary staff working in the NHS, and private sector health care organizations providing services to the NHS. Given that the Code has changed in content over the years, any assessment of the impact of DH intervention on international recruitment activity must also take account of these four points in the timeline: 1999, 2001, 2003 and 2004.

There is an assumption made by some commentators, both in the United Kingdom and elsewhere, that the Code sets out to "prevent" all international recruitment from low-income countries. It is important to stress that it was not intended for this purpose. The Code aims to prevent "active" recruitment initiated by the NHS in England, and targetted at specified countries . It is directed at NHS employers in England, recruitment agencies commissioned to recruit staff on behalf of NHS employers, temporary staffing agencies providing staff to NHS employers, and private sector employers in England, particularly if they are providing NHS-funded care (England is the largest of the four countries in the United Kingdom; devolved government means that each of the four countries in the United Kingdom has policy responsibility for NHS workforce issues, but some aspects of immigration and regulation policy are retained at the level of the United Kingdom. Scotland has also issued a similar Code) [6].

The Code also sets out guidelines for good practice on international recruitment for United Kingdom employers, covering aspects of recruitment, selection, induction and equal opportunities in employment, pay and career prospects. NHS employers in an earlier study reported that they found the 2001 version of the Code helpful in directing their recruitment to be effective and "ethical" [7]. Some surveys of internationally recruited nurses have reported, however, that some nurses feel they have been discriminated against when being graded for pay levels [8].

In addition to so-called "active" recruitment, various types of "passive" recruitment have contributed to increasing the number of international health workers coming to the NHS in England. In the context of the Code these are not regarded as "active" recruitment, and so are not taken to contradict or undermine the Department of Health Code:

• Some international staff initiate recruitment by applying for employment in the United Kingdom while located abroad (increased access to the Internet has made this easier).

• Some staff will move to the United Kingdom initially for educational and training purposes, and then be recruited when they are in the United Kingdom.

• Some "international" workers will already be resident in the United Kingdom but not yet in employment – such as refugees.

• Some health care workers will be recruited initially by private-sector health care employers in England who may not be bound by the Code, but these workers may move quickly to NHS jobs once they have arrived in the United Kingdom. This practice does not break the Code, as the workers were recruited by the NHS when they were in England, but it has been termed "back-door" recruitment [8].

These issues of coverage and content require careful consideration when assessing whether the Code has met its objectives. It should also be noted that an additional problem is caused by that fact that the NHS in England did not conduct standard or systematic central monitoring of the numbers of all international health professionals it recruited. The NHS is the main, but not the only, source of employment for health care professionals. While it is possible to monitor and track "inflow" of health professionals to the country (the United Kingdom) using work permit and professional registration data, it is not possible to identify which of these health professionals – notably nurses – have been actively recruited directly by the NHS in England, and thus differentiate those who have come to the United Kingdom to work for other (i.e. non-NHS) employers or for education purposes.

## Methods

The study comprised an analysis of professional registration data and work permit data in the United Kingdom to examine trends in "inflow" of doctors and nurses from other countries, and country case studies in two countries with long-term migratory links with England: Ghana and Kenya.

Registration data from the General Medical Council (GMC) and the Nursing and Midwifery Council (NMC) were examined. All doctors and nurses who wish to practice in the United Kingdom must be registered with the relevant United Kingdom body, which enables estimates to be made of the annual number of "new" registrants from other countries. The main constraint on interpretation of the data is that the data show only that the individual has been registered: the individual may not have actually moved to or begun working in the United Kingdom. As noted above, the data also do not enable an assessment of the employment destination, NHS or otherwise.

The second source of information was the inflow data on applications for work permits. Most non-United Kingdom applicants for employment from countries outside the European Union/European Economic Area (EU/EEA) who wish to take up employment in the United Kingdom are required to obtain a work permit. Work permit data can therefore be used as another source of information on trends in inflow from non-EU/EEA countries. Work permits are issued for a specified period of time for work in the United Kingdom.

Professional registration data and work permit data were used as the best proxy measures because, as noted above, there is no systematic and standard national monitoring by NHS England of active international recruitment of nurses.

In assessing the impact of the Code, it must be borne in mind that there is no single date to focus on as the benchmark or start date. As described above, there have been different versions of guidelines and Codes in use since 1999. It should also be noted that these data record individuals applying to enter the United Kingdom to work as health professionals. If a nurse leaves an African or Asian country but works as a care assistant in the United Kingdom, this may not be recorded by these data as a nurse "moving" from one country to the other.

## Results

Registration data were analysed to identify how many doctors and nurses had registered in the United Kingdom from different types of countries: countries on the "list" of those from which the NHS was not supposed to actively recruit; high-income countries; and the low-income countries "exempted" from the list at the request of their governments (China, India and the Philippines). Tracking the numbers and relative proportion of health professionals being registered from "list" countries was one way of evaluating the impact of the Code.

**Figure 1:**
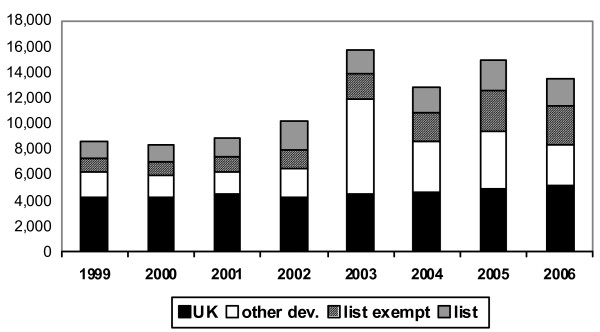
**Doctors: new GMC full registrants from the United Kingdom, other developed countries, list-exempt countries and list countries, 1999–2006**. Source: General Medical Council, United Kingdom.

Figure 1 presents the registration data for doctors. The "spike" in registration in 2003 is reportedly an artefact rather than an indicator of a true increase in inflow. It occurred as a result of changes to the Medical Act (Statutory Instrument 2002//3135) that were to come into effect as a cut-off point in the United Kingdom at the end of 2003 and accelerated precautionary applications from graduates of certain universities in specified places, such as Hong Kong and Malaysia: they were registering even if they had no current plans to move to the United Kingdom. Setting this aside, there has been little change in the annual number of registrants from the "list" countries, which has varied between 1800 and 2200 across the period under consideration.

**Figure 2:**
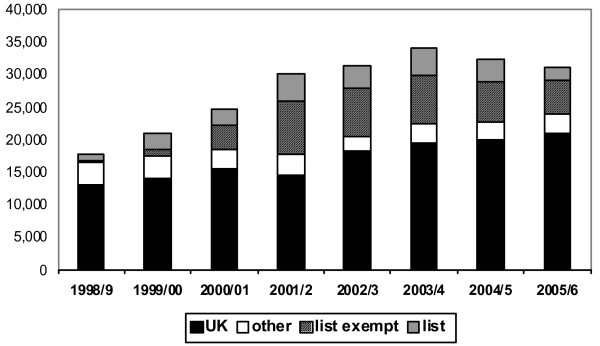
**Nurses: new registrants from the United Kingdom, other developed countries, list-exempt countries and list countries, 1998–2006**. Source: Nurses and Midwives Council, United Kingdom.

Figure 2 presents the registration data for nurses, collated in a similar format. The peak year for nurse registrants from overseas sources was 2001–2002. In the period between 2001–2002 and 2005–2006 the annual number of nurses registering from "list" countries declined by more than half and the annual number from list-exempt countries by more than one third, while the annual number registering from United Kingdom sources increased significantly.

The second source of data was on work permits. Work permit data are not directly comparable with registration data. They cover different calendar years, do not cover individuals from EU countries, and provide data only on non-United Kingdom source countries, so cannot be used to assess the relative contribution of "new" United Kingdom sources in overall numbers of new entrants to the labour market. They do, however, provide an alternative measure of the inflow of health professionals from non-EU countries.

**Figure 3:**
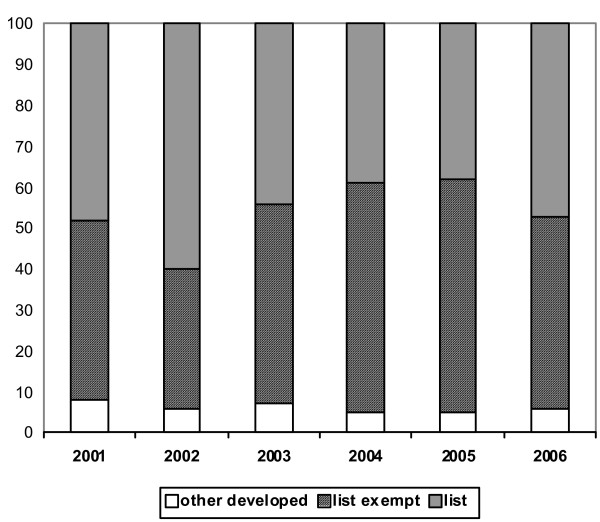
**Work permits issued to doctors: percentage by type of country, 2001–2006**. Source: Work Permits, United Kingdom.

Figure 3 presents the data on work permits, shown in percentages by category of source country. The number of work permits/first time approvals issued to doctors applying for the first time to work in the United Kingdom increased rapidly from 1999 (547) to 2004 (2645) and then declined to 1931 in 2006 The data on the allocation of work permits to doctors that are illustrated in Figure 3 show some fluctuation between "list" and list-exempt countries over the period 2001 to 2006, but no overall trend of change. There has been no sign of a relative decrease in the percentage of approvals for applicants from "list" countries in the period under consideration.

**Figure 4:**
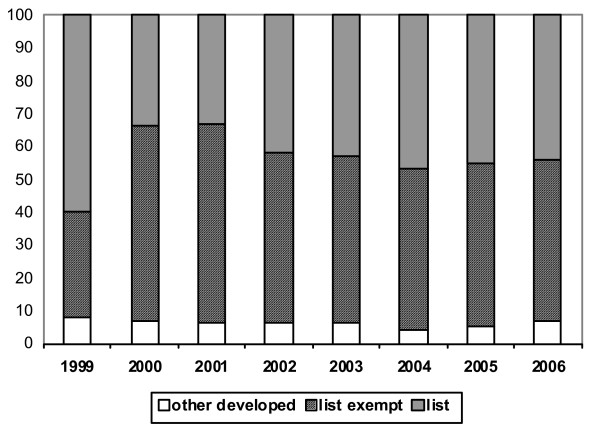
**Work permits issued to nurses: percentage by type of country, 1999–2006**. Source: Work Permits, United Kingdom.

The overall annual number of work permit/first permission approvals issued to nurses increased rapidly – from 1918 in 1999 to 15 246 in 2002 – and then declined to 10 730 in 2005, with a further marked decline in 2006, to 4931. The annual percentage distribution across different types of source country is shown in Figure 4. As with the data on permits for doctors, there is little sign of any marked trend of change in the percentage of nurses from list countries and from list-exempt countries being issued with work permits over the period 2001–2006.

### Country case studies

The other source of information for the study was data generated by case studies conducted in Ghana and Kenya. These case studies were conducted to ascertain the current outflow of health professionals to the United Kingdom and to other countries, in order to assess the relative significance of the United Kingdom as a destination for health professionals and to assess the "visibility" of the DH Code as a policy instrument in the health workforce policy and planning context in these countries.

Both country case studies relied on three methods of data collection: document and secondary data review, key informant interview and group discussion. Key informants were selected from representatives of the main organizations involved in human resource issues in the country. These included professional associations and regulatory bodies, ministry of health officials with responsibility for human resources, training institutions and development projects focused on human resource development issues. Group discussions were held with health professionals and trainees. Documents and secondary data reviewed were those that identified the scale of health professional migration. A total of 35 key informants were interviewed and three group discussions held.

### Kenya

The data suggest that Kenya suffers from an acute shortage of nurses, yet there are reports of nurse unemployment [9]. This apparent contradiction reportedly has arisen because the Kenyan health system does not fund employment of sufficient nurses to meet its identified need.

An emergency hire programme supported by international aid and the Government of Kenya recruited an estimated 3000 nurses, mostly to serve in rural health facilities of both the government and faith-based organizations in 2006 (see [10] for an update). There is unequal distribution of the health care labour force between urban and rural areas and especially remote parts of the country, which has been driven by deployment procedures and relative changes in reimbursement packages, between those offered by the public sector and those offered by the faith-based organizations that disproportionately serve rural areas.

Table 1 presents data compiled by the Nursing Council of Kenya on the number of nurses whose qualifications were verified between January 1993 and December 2006. Nurses require qualification verification as part of the registration process in a second country.

**Table 1:** Kenya: Total number of nurses verified to have applied for foreign registration, January 1993 to December 2006

**Country**	**1993**	**1994**	**1995**	**1996**	**1997**	**1998**	**1999**	**2000**	**2001**	**2002**	**2003**	**2004**	**2005**	**2006**	**Total**
UK	20	15	16	20	32	32	80	199	687	210	253	324	158	72	2118
USA	6	5	4	10	16	40	46	45	174	356	656	263	255	220	2096
Others	8	23	30	b26	31	29	25	42	52	26	31	56	78	98	555
Total	34	43	50	56	79	101	151	286	913	592	940	643	491	390	4769

Source: Kenya Nursing and Midwives Council. NB: Totals have been adjusted to add correctly. The table provided cites the total number of nurses verified over the period as 4783.

There were peaks in the numbers seeking verification for exit to the United Kingdom in 2001 and exit to the United States of America in 2003. For both countries, verification numbers have fallen sharply since their peaks. That there have been sharp falls in exit verification for both the United Kingdom and the United States does not support an explanation based on the impact of United Kingdom recruitment policy alone: the United States does not have a code or restriction on recruitment, yet numbers applying to the United States have also fallen.

The only internal source of data on doctor migration is the numbers seeking a "Certificate of Good Standing" from the Kenya Medical and Dental Association. This is required for doctors planning to practise outside the country or to go abroad for postgraduate medical training. A register of these shows that 28 doctors had sought a letter since February 2007, with three more doctors in the process of application.

Training is the usual vehicle by which doctors migrate: it is reported to be relatively rare for a doctor to migrate directly to an overseas post other than a training post, or to set up an overseas private practice.

Stakeholders in Kenya provided multiple explanations of the trends observed in relation to the flows of health professionals to the United Kingdom, few of which appeared to make a direct connection to the Code of Practice. Better pay and conditions for doctors in Kenya and to a lesser extent for nurses were highlighted, but the main factor identified was greater difficulties in achieving access to the United Kingdom labour market. Informants in Kenya reported that this was due to greater difficulties in obtaining visas, more stringent United Kingdom professional "adaptation" requirements and difficulties in securing United Kingdom clinical placements, and increased total costs of the process.

Many respondents in Kenya commented on past history in the country of very active recruitment on behalf (direct or indirect) of the United Kingdom NHS. They remembered seminars in hotels, visiting agents, and newspaper advertisements. Respondents reported that these activities were not now occurring on behalf of the United Kingdom. One reported example was a recruitment agency in Kenya that had in the past supported many nurses who wanted to migrate to the United Kingdom: it stated that it would no longer advise nurses to consider the United Kingdom but would point them in the direction of the United States or Australia, instead. It cited problems related to increased difficulty of access to the United Kingdom as the reason for this advice.

Few accounts of unscrupulous recruitment agent activity were reported in Kenya. This was reportedly mainly a problem associated with the recruitment of the unskilled labour force. However, it was perceived as unethical to recruit qualified professional nurses for relatively unskilled jobs in nursing homes and the like. A number of respondents were conscious that Kenyan nurses had found themselves working in such jobs and this was considered demeaning and exploitative.

### Ghana

The Ghana Health Services estimated that there were 1446 doctors and 14 507 nurses employed in 2006. Longer-term trends for numbers of nurses and midwives in Ghana (1999–2005) provided by the Nurses and Midwives' Council for Ghana show that the numbers of registrants have increased considerably over the nine-year period. The numbers of unemployed nurses and doctors in Ghana were reportedly estimated to be insignificant.

**Figure 5:**
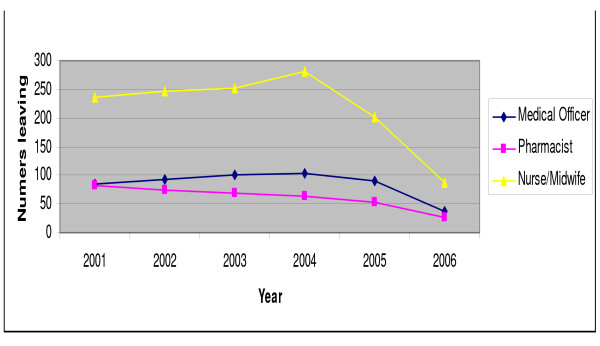
**Loss of staff from individual public, CHAG and military health facilities, by cadre**. Source: Ministry of Health, Ghana.

The Ministry of Health, which includes the public services of the Ghana Health Services and private and military sector services, estimates the total loss of staff from the public, Christian Health Association of Ghana (CHAG) and military health facilities (Figure 5). These data indicate that staff losses have reduced since 2004, having shown an increase between 2001 and 2004 for nurses/midwives and medical officers. Data also show that "vacation of post" – the usual description for migration out of Ghana – has declined as a cause of attrition. It should be noted that these total movements of health workers from individual health facilities will include some who move between facilities, rather than being lost to the system.

Additional information on outflow to other countries was obtained from health professional bodies. Data provided by the Pharmacy Council showed that the number of pharmacists requesting letters of confirmation of qualifications reached a peak in 2003 and had stabilized at about half their peak levels between 2004 and 2006. Data provided by the Ghana Nursing and Midwives Council show a declining trend in rate of requests for verification of qualifications from a peak in 2003, with an overall 34.5% reduction between 2003 and 2005. The dominant target destination in recent years had been the United Kingdom (71%), followed by the United States (22%). The Nursing and Midwives Council further reported that validation requests had fallen to 56 in 2006, from a figure of 686 in the previous year. This rapid decline was attributed by respondents to two main factors: Government of Ghana policy changes and international labour market changes.

The Government of Ghana has put in place a number of policy measures designed to reduce the rate of migration of health professionals:

• improved pay and conditions packages of doctors and other health professionals.

• new procedures that make it more difficult to evade the provision of the bond by which nurses trained with public funds are required to work for five years for the Ministry of Health or repay the cost of training.

For doctors, similar measures to enforce a bond policy with similar intentions have not been taken, but there had been an extension to the period working as a house officer, and the Ghana College of Physicians and Surgeons had been instituted in 2003 to expand provision of postgraduate medical training.

International market developments have had also reportedly had significant impact on migration trends. Increasing barriers to entry to the United Kingdom were seen by most respondents in Ghana as the most important explanation of the decline in migration trends. These included:

• greater difficulties in obtaining visas and jobs;

• more stringent "adaptation" requirements and difficulties in securing placements;

• a perception of difficult conditions in the United Kingdom NHS.

Respondents noted that the dominant mode of recruitment of health professionals to the United Kingdom has been by word of mouth and operating through collegiate networks. None of the respondents in Ghana believed that the Code of Practice had produced a significant effect, and they attributed recent reduced international recruitment activity to the reduced employment opportunities in the United Kingdom.

## Discussion

As noted earlier, some policy analysts have misunderstood the actual content and coverage of the DH Code. It is important to stress the key points, in terms of the content and objectives of the Code:

• The Code places restrictions on active recruitment by the NHS; it does not aim to prevent recruitment of other kinds (e.g. movement for education).

• The Code provides a list of countries that should not be targeted for active international recruitment; this list was only made available some time after the Code was published in 2001, and is subject to review.

• The Code does not cover the whole of the United Kingdom; it covers primarily the NHS in England, which is the main but not the only employer of health care professionals in the country.

• The Code includes information on good practices for NHS employers on how to conduct effective and so called "ethical" international recruitment.

Secondly, it must be highlighted that there was no systematic or comprehensive monitoring of "active" international recruitment, so data are not publicly available to test the effect of the Code in detail, in terms of changes in flows from different types of source country. It is not possible to identify in detail the actual number of health care professionals who were recruited by the NHS, or which proportion of this group had been "actively" recruited. The absence of systematic monitoring of NHS international recruitment activity means that any evaluation has to rely on proxy measures related to professional registration and to work permits issued. Neither measure is ideal or provides a complete measure of inflow.

It is clear that available data do show a considerable recent reduction in inflow of health professionals to the United Kingdom for the period during which the Code has been implemented and strengthened, from the peak years of inflow up to 2002 (for nurses) and 2004 (for doctors). But it is important not to ascribe these changes only – or perhaps even mainly – to the Code. There are multiple reported causes of this recent decline, including declining demand in the United Kingdom and the introduction of more stringent registration and entry requirements. Furthermore, the trend data alone are not sufficient to demonstrate causality in relation to any one policy instrument.

The case studies in Kenya and in Ghana also highlighted recent apparent reductions in outflow of nurses and doctors, but this was in part reportedly a result of relative improvements in working conditions in the countries, and was also attributed (in the case of declining flows to the United Kingdom) to tougher entry requirements and a reduction in demand from the United Kingdom.

There was little reported knowledge of the Code in the case study countries, and some misunderstanding about the extent to which the Code was, or could be, responsible for the increased difficulty in gaining access to the United Kingdom health labour market in recent years. Dissemination and communication of the contents of the Code to relevant parties within the case study countries had apparently been largely absent.

## Conclusion

The DH Code is a single country instrument, and it has been applied in a country where there is considerable scope for compliance and control because so much health sector employment activity is located within the public sector NHS, and where there was in essence a "single point of entry" for international recruits – via one point of professional registration and one point of work permit application. These can be regarded as supportive conditions in which to apply and monitor a government-led Code.

However, the potential to assess the effect of the Code in England is constrained by the limitations in available databases. This is a crucial lesson for those considering a global code: without a clear link between explicit objectives of a Code, and relevant monitoring capacity, it is not possible to judge the actual impact of a Code.

A second message for policy-makers is that attempts to use a single country code in other countries where there are a multiplicity of independent, private-sector health care employers, or where there is a federated political and regulatory structure, will be a much more challenging and complex issue.

Finally, there is a message about the importance of the "visibility" of any recruitment Code – among policy-makers, employers and potential recruits. The DH Code has a good level of recognition in the NHS, but would benefit from better dissemination in low-income countries, particularly in Africa, together with further consultation on the appropriateness of its provisions in specific countries. To achieve high visibility and recognition of any global code will be a much bigger challenge.

## Competing interests

The authors declare that they have no competing interests.

## Authors' contributions

JB contributed to study design, analysed the United Kingdom data and edited this paper. BMcP contributed to the design of the study and edited country case study reports. KM conducted a country case study, as did GR.
